# Heuristic test reveals little effect of learning and maturation on early prey capture experiences in a web-building spider

**DOI:** 10.1038/s41598-024-61252-7

**Published:** 2024-05-22

**Authors:** Madison A. Rittinger, Rafael L. Rodríguez, Ignacio Escalante

**Affiliations:** 1 Behavioral and Molecular Ecology Group, Department of Biological Sciences, University of Wisconsin-Milwaukee, Milwaukee, WI USA; 2 Escuela de Biología, Universidad de Costa Rica, Cuidad Universitaria Rodrigo Facio, 2060, San José, Costa Rica; 3 Present Address: Department of Biological Sciences, University of Illinois Chicago, Chicago, IL USA

**Keywords:** Ecology, Psychology, Zoology, Ecology

## Abstract

Behaviors can vary throughout an animal’s life and this variation can often be explained by changes associated with learning and/or maturing. Currently, there is little consensus regarding how these processes interact to affect behaviors. Here we proposed a heuristic approach to disentangle the effects of learning and maturation on behavior and applied it to the predatory behaviors of *Physocyclus globosus* spiderlings. We varied the degree of prey difficulty and familiarity spiderlings received along the first instar and across the molt to the second instar and quantified the time spiderlings spent wrapping prey, as a proxy for prey capture efficiency. We found no overall evidence for learning or maturation. Changes in efficiency were mainly due to the switch from difficult to easy prey, or vice versa. However, there was one treatment where spiderlings improved in efficiency before and after the molt, without a switch in prey type. This provides some indication that difficult prey may offer more opportunity for learning or maturation to impact behavior. Although we found little effect of learning or maturation on prey capture efficiency, we suggest that our heuristic approach is effective and could be useful in investigating these processes in other behaviors and other animals.

## Introduction

Many behaviors change as animals develop. Along their ontogeny, animals acquire knowledge through experience with many behaviors (i.e., learning)^1–3^. Many animals use learning to adaptively adjust their behavior^1,2^. Simultaneously, animals undergo the growth and development of sensory, motor, and nervous systems as they develop (i.e., maturation)^2,4,5^. The development of these systems and structures during maturation can subsequently affect behavioral processes ^6–9^. Consequently, both learning and maturation can impact behaviors, yet we do not fully understand how these processes interact. In some instances, learning is predicted to precede maturation^10^, and learning seems to be the most important process in determining behaviors^11^. In other instances, maturation is thought to precede or work simultaneously, or synergistically, with learning^12–14^.

Here we propose a novel heuristic approach for when to expect stronger contributions from learning or maturation on behavior. We also provide an empirical criterion for how to distinguish between their effects. We reason that learning should be most likely to influence behaviors regarding improvement in skill cf^10,15^. By skill, we refer to improvements in behavior resulting from making correct decisions or increased fine motor control due to repeated exposure to a task. In contrast, maturation should be most likely to influence behaviors regarding improvement in strength throughout development. By strength we refer to improvements in behavior resulting from increased muscle mass and hydrostatic forces. Albeit blunt, this distinction offers an empirical criterion for distinguishing the roles of learning and maturation.

We frame our heuristic approach in animals whose ontogeny involves discrete maturational events (e.g., molting in arthropods). The effects of learning should be easiest to detect within maturational events, and the effects of maturation across those events. Across maturational events like molting, there is a significant increase in body size^7,16^, and therefore the effect of maturation should be most evident here. However, the effects of learning do not require growth and should be most evident before any confounding morphological changes occur. This is not to say that the effects of learning are not present across, and the effects of maturation within, maturational events. Rather, we are predicting where the effects of these processes can best be distinguished.

We implemented the above heuristic by exploring the early development of predatory behaviors in spiderlings. Predatory behaviors presents an ideal opportunity to investigate the effects of learning versus maturation as they are critical for a predator’s survival and hence, are repeated starting at a young age^17–19^. The effects of learning can be disentangled by accounting for prior experience and the effects of maturation by controlling the developmental stage during prey capture.

Learning and maturation have a complex role in the development of predatory behaviors in arachnids. On the one hand, learning affects predatory behaviors in spiders. Individuals with prior experience navigate their webs more efficiently^20^; build webs that are more effective at capturing prey^13,21–23^; and form a preference for familiar prey types^22^. On the other hand, maturation also affects spider predatory behaviors. Molting is a major step in maturation in spiders. Individuals undergo multiple molts in which they increase in body size, leg length, chelicerae size, and silk thickness^7^. These changes associated with maturation affect web-building behavior^18^; distance required to attack prey^13^; number of attacks^24^; and the time required to subdue prey^24,25^.

We explored the effects of learning and maturation on spiderling prey capture efficiency. To disentangle the effects of these two processes, we manipulated two variables: the degree of familiarity and difficulty of the prey spiderlings received. We manipulated these variables by using two different prey items: an easy prey item to capture (a *Drosophila melanogaster* fruit fly) or a difficult prey item (a *Paratrechina longicornis* [Formicidae] worker ant). For this experiment, we expected that any improvements in behavior due to learning will be most easily discernable before molting, while any improvements due to maturation will be most easily discernable across a molt.

Specifically, if learning primarily impacts prey capture efficiency, we predicted that spiderlings should improve their prey capture performance once they have experience with prey before a molt. This improvement could be greater after switching from difficult to easy prey within the instar; e.g., if experience with difficult prey offers an enhanced learning experience^26,27^. By contrast, if maturing primarily impacts prey capture efficiency, we predicted that spiderlings should improve their prey capture performance across the molt to the second instar, regardless of prey familiarity. The magnitude of this improvement could depend on the type of prey spiderlings received before the molt; e.g., if difficult prey struggle more, which may promote muscle development. If learning and maturation both impact prey capture efficiency, we predicted that spiderlings should improve their prey capture performance after experience with prey before and across the molt. Note that in our experiment, the switch in prey difficulty alone could account for changes in behavior. Specifically, the difference in ease of capturing the prey could produce an apparent improvement in behavior without any learning or maturation; e.g., after switching from difficult to easy prey or vice versa switching from easy to difficult prey. If so, we predicted the magnitude of change in behavior will be similar, regardless of whether the switch occurred before or across a molt. In all scenarios, we expected spiderlings to capture easy prey more efficiently than difficult prey, regardless of experience.

## Materials and methods

### Study species 

We worked with *Physocyclus globosus* (Taczanowski 1874) (Pholcidae, Araneae) cellar spiders. These spiders are common in manmade structures^28–30^ and build dome-shaped irregular sheet webs that capture a variety of prey starting at a very young age^29,31,32^. Pre-nymph spiderlings remain close to their mothers after hatching, and, after ~ 10 days, spiderlings molt to the first instar, disperse to build webs, and begin to capture prey^17^. Web-building behavior, and likely prey capture, varies across instars of *P. globosus*^31,33^. In total, there are 7–9 instars until adulthood in this species^31^.

We collected adult male and female *P. globosus* in one building on the Universidad de Costa Rica campus in San José, Costa Rica. We kept adults in the lab at ~ 20 °C and 80% humidity for several days before they were mated. We randomly paired adults (one male with one female), and then placed that male on the female’s web to allow them to mate. We only used spiderlings from one egg sac from each mated pair. Thus, each clutch of spiderlings came from a different male/female pair, and there were likely few, if any half- siblings across clutches (although we do not know the prior mating history of females).

After the eggs hatched, we individually placed pre-nymphs in plastic 50 ml round cups (4 cm tall, 3 cm upper diameter, and 2.5 cm base diameter). The inner walls and the floor were covered with bond white paper so the spiderling could walk and attach threads. The cup was covered with cling wrap with a small (0.5 cm) longitudinal opening to introduce the prey. We began trials 10 days after placing spiderlings in their individual cups. During this period, the spiderlings molted to the first instar and built their first prey capture webs^33^. By using spiderlings in their first instar and across the molt to the second instar, we ensured that we fully controlled their early experience with prey—we can be certain that they had no prior experience capturing prey outside our treatments. We could not physically identify the sex of spiderlings at this stage^31^.

No approval of a research ethics committee was required for this research. However, a research proposal was approved by the Sistema de Estudios de Posgrado of the Universidad de Costa Rica. We continuously monitored spider welfare and provided ideal rearing conditions throughout this study to ensure all individuals were treated as humanely as possible.

### Prey capture behavior

The attack behavior of *P. globosus* and other pholcids includes four main behavioral stages or modules that summarize 11 distinct behaviors: (i) detecting prey and initial attack, (ii) wrapping, (iii) handling, and (iv) biting^17,34–37^. A spider detects prey as it contacts the web, then approaches and touches it. Sometimes the spider quickly pulls the prey from the substrate onto the web (e.g., if the prey is not fully in the web yet). Then the spider wraps the prey, applying silk lines with alternate movements of the hind legs (the distal-most pairs of legs)^17^. Next, in the handling phase, the spider cuts and attaches new threads of silk around the prey and moves it to the sheet (i.e., the main section of web) if it is not already there. Finally, the spider gives approximately a dozen short bites to inject digestive enzymes and venom before settling to feed for a long period of time^7,17^.

In this study, we used the total time spent wrapping to quantify prey capture efficiency (for a detailed description of how we quantified this, see Behavioral Trials). In this species, wrapping prey in silk is essential for prey capture; restrains the movement of prey; and explains the majority of behavioral variation when prey vary in difficulty^17^. Note that time spent wrapping offers a "less is more" measure of prey capture efficiency, with less time wrapping corresponding to higher efficiency. Additionally, time spent wrapping prey could be indicative of both skill and strength. Spiderlings could learn to wrap prey more efficiently and/or may have more ease handling prey during wrapping after increased muscle growth through maturation. Therefore, not only is time spent wrapping one of the most vital behaviors for prey capture in this species, but it also provides an ideal behavioral metric for our proposed heuristic.

### Behavioral trials

Ten days after emergence, spiderlings had already molted, built a sheet web, and were hanging upside down in the center of the sheet. We fed them their first prey item at this time. We gave each spiderling one prey every three days, for a total of three prey items during this instar. We then gave them their fourth prey after the molt to the second instar, seven days after the third prey. This timeline was based on pilot data that showed that most spiderlings were motivated to attack three prey in the first instar. Controlling the amount of prey spiderlings received ensured similar motivation to attack prey in all trials.

We used forceps to place prey directly on the center of the spiderling’s web, through the covering on each cup. We then recorded the attack using a SONY HandiCAM DCR-VX 1000 camera with three macro lens (+ 4 X each) at 30 frames per second speed. We analyzed the videos with the software Etholog 2.2 (Ottoni 2000) to obtain the total time spent wrapping prey. We quantified time spent wrapping in each video by observing, identifying, and manually noting the exact frame in which we first saw the start and end of a wrapping bout. While wrapping, the spider rapidly alternates the distal-most pairs of legs, pulls silk from their spinnerets, and moves its abdomen sideways^17^. Observing these behaviors allowed us to quantify the exact duration of one wrapping bout. We visually marked the start and end of each of the many (> 20) wrapping bouts throughout the attack. We added the duration of all wrapping bouts to report the total time each spiderling spent wrapping a given prey in each trial.

In each trial, we gave spiderlings difficult or easy prey. The difficult prey was a *P. longicornis* worker ant (hereafter “ant”). The easy prey was a *D. melanogaster* fruit fly (hereafter “fly”). We aimed to standardize the size of prey, with both measuring approximately 2.5 mm long. Ants and flies are common prey for pholcids^28,37,38^. Spiderlings, therefore, should be equally motivated to attack both species. Ants are considered difficult prey to subdue for several spider species^13,17,35,39–43^. For *P. globosus* in particular, ants move more while being attacked and can damage the legs of spiderlings^17^. In this study, spiderlings took longer to wrap difficult prey (see Results).

### Prey sequence treatments

**Figure 1:**
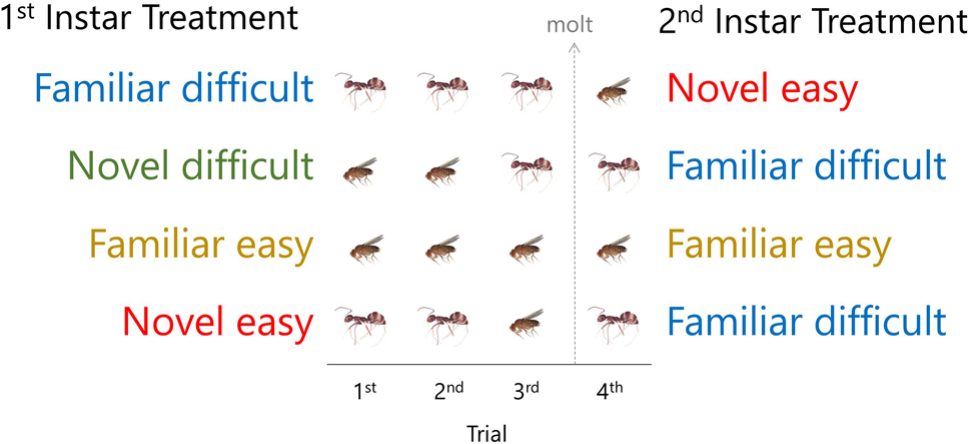
Experimental design to assess the roles of maturation and learning in the predatory behavior of *Physocyclus globosus* (Pholcidae) spiderlings. Trials 1–3 were during the spiderling’s first instar, while trial 4 was after the molt (denoted by the dashed line) to the second instar. Treatments differed in the sequence of prey given to spiderlings in their first and second instar. For the first instar, treatments are defined by the type of prey given to spiderlings in their third trial, relative to prior trials. For the second instar, treatments are defined by the type of prey given to the spiderlings in their fourth trial (after the molt), relative to the third trial. Difficult prey were ants, and easy prey were fruit flies.

We randomly assigned spiderlings from ten broods (mean ± SD = 11 ± 9 spiderlings per brood) to the different prey sequence treatments. We made sure to include equal numbers of spiderlings from each brood in each treatment, as much as we could. Some broods yielded odd numbers of spiderlings (or non-multiples of 4 even numbers). Treatments differed in whether spiderlings received familiar or novel prey and whether that prey was easy or difficult. In the first two trials, each spiderling received the same prey, either easy or difficult. This offered an opportunity for learning to occur. In the third trial, we switched the type of prey given to half of the spiderlings. This created a full factorial design for the switch in prey type between the spiders’ second and third trials. There were four first-instar treatments in which spiderlings received a third prey item that was either familiar and difficult, novel and difficult, familiar and easy, or novel and easy (Fig. 1).

After the molt to the second instar, we switched the prey (based on what was given in the third trial) for half the spiderlings. This created a full factorial design for the switch in prey type across the molt. There were three second-instar treatments in which spiderlings received prey that was either novel and easy, familiar and difficult, or familiar and easy. We note that there are other possible combinations across the molt that we did not implement (e.g., a novel difficult treatment prey in the fourth trial). However, our goal was to create a full-factorial design of switches in prey type before and across the molt after an initial experience with prey, which our experiment offers.

### Statistical analysis

We ran all analyses with JMP Pro v. 16.0.0 (SAS Institute Inc., Cary, NC). We used linear mixed models with total time spent wrapping (log_10_-transformed) as the response variable (for raw data, see Supplementary Materials). We used the log_10_ transformation of time spent wrapping to meet the assumption of normality (Anderson–Darling Test: *p* = 0.58).

We tested the predictions of all of the hypotheses with models that had the following explanatory terms: trial number (as an ordinal variable); prey sequence treatment (the treatment before the molt on the left, and after the molt on the right Fig. 1); and the interaction between trail number and treatment. The term for trial number tests for an overall change in prey capture efficiency, regardless of the familiarity and difficulty of prey. The term for treatments tests for the effect of familiarity and difficulty of prey on capture efficiency. The interaction term tests whether the effect of experience on prey capture efficiency varied with treatment. We also included brood and individual spiderling identity nested within brood as random effects in the models.

To test the prediction of the learning hypothesis—that spiderlings should improve their efficiency within an instar after experience with prey— we fit the above model using data only from trials along the first instar (1–3). To test the prediction of the maturation hypothesis— that spiderlings should improve their efficiency across the molt to the second instar, regardless of prey familiarity— we fit the model using data only from trials directly before and after the molt (3 & 4). We used this subset of data to focus on where the effects of maturation should be most evident (across the molt) and excluded data where learning should be most evident (along an instar).

The above two tests each included data from trial 3. We therefore adjusted our criterion for significance to *α* = 0.05/2 = 0.025 following the Bonferroni method to avoid the risk of spurious significance^44^.

To test whether the switch in prey difficulty alone was primarily responsible for changes in prey capture efficiency, we compared similar switches before and across the molt. We reasoned that if the switch in prey type primarily impacts efficiency, the changes in this metric should be of similar magnitude (or “steepness”) regardless of whether they occurred before or across the molt. We thus ran two directed post-hoc analyses. In the first model, we only included data for switches from difficult to easy prey (one switch occurred before and one across the molt). In the second model, we only included data for switches from easy to difficult prey (one switch occurred before and one across the molt). In these models, the explanatory terms were as above, and the trial × treatment interaction tests for a difference in the "steepness" of the change in efficiency according to whether the switch occurred before or across the molt.

A portion of this dataset (trial 1) was used in another study that describes the interaction between the behavior of *P. globusus* spiderlings and the behavior of their prey on the spiderling’s first prey capture experience^17^.

## Results

**Figure 2:**
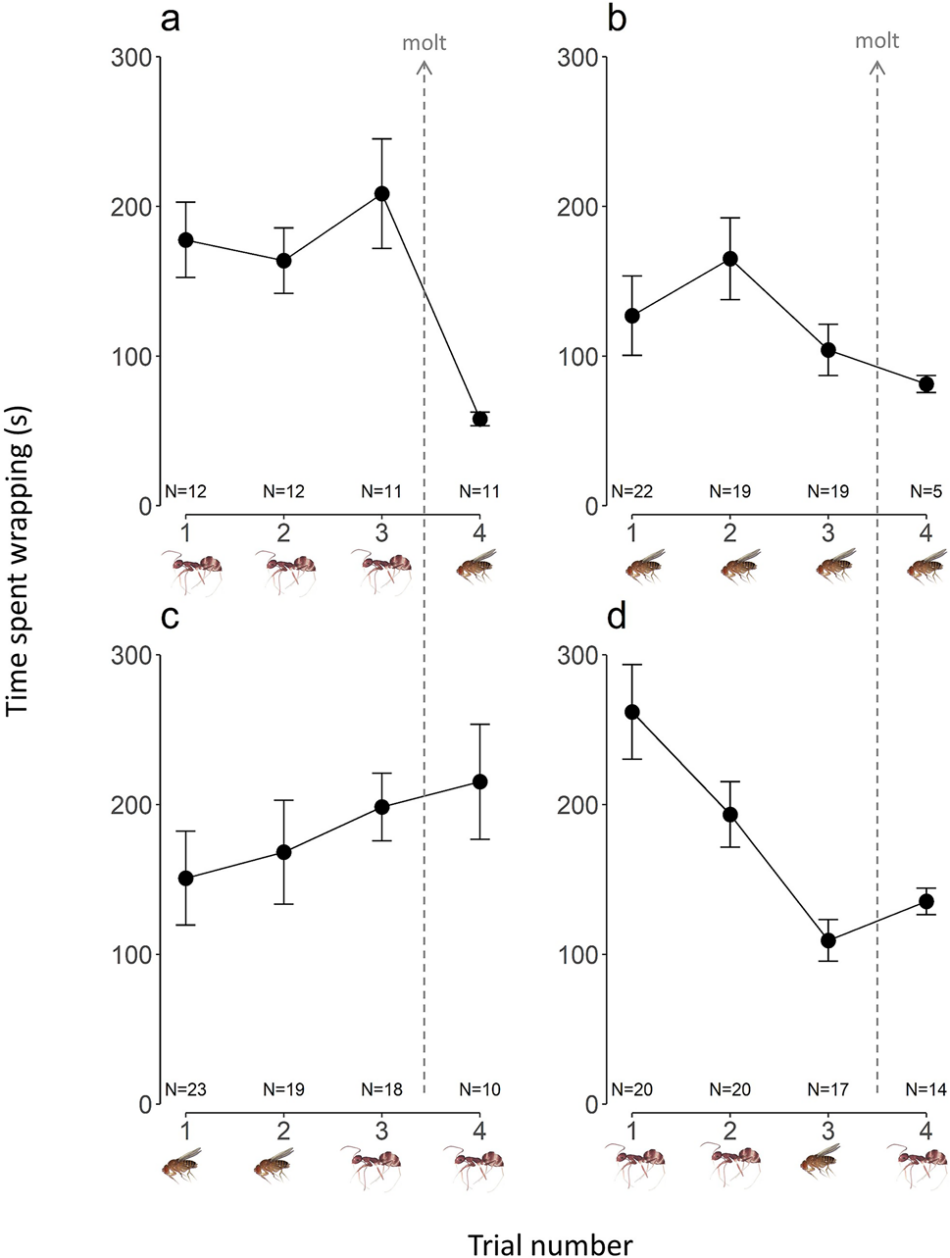
Mean ± SE of total time *P. globosus* (Pholcidae) spiderlings wrapped prey across four trials. We use total time spent wrapping as a proxy for prey capture efficiency; with less time spent wrapping indicating an improvement in efficiency. Spiderlings were randomly assigned a prey-sequence treatment (pictured under trial number) that varied in prey familiarity and difficulty (ants are difficult and flies are easy to capture). Trials 1–3 were during the spiderling’s first instar while trial 4 was after the molt (denoted by the dashed line) to the second instar. In trials 1 and 2, all spiderlings received the same prey, but we changed the prey type offered to half of the spiderlings in trial 3. This created four treatments within the first instar in which spiderlings received a third prey item that was either (a) familiar and difficult, (b) familiar and easy, (c) novel and difficult, or (d) novel and easy. After the molt to the second instar, we gave spiderlings a fourth prey item that was either (a) novel and easy, (b) familiar and easy, or (c and d) familiar and difficult. Sample sizes vary across trials because some spiderlings did not complete all four trials; e.g., due to mortality.

Overall, spiderlings took longer to wrap difficult prey (ants: mean ± standard error [SE] = 198.4 ± 9.9 s) than easy prey (flies: 129.4 ± 9.9 s) (F_1_, _185.7_ = 31.41, *p* < 0.0001, Fig. 2). There was no overall improvement in prey capture efficiency along the first instar (non-significant main term for trial in Table 1; Fig. 2). However, there was improvement along the instar for one treatment (significant treatment and treatment x trial interaction terms in Table 1; Fig. 2). Specifically, the treatment where spiderlings were familiar with difficult prey and then received easy prey improved in efficiency (Fig. 2d). Interestingly, spiderlings that received difficult prey in trials 1 and 2 improved in efficiency in some cases (Fig. 2d, trials 1 and 2) but not others (Fig. 2a, trials 1–3). There was variation in efficiency with difficult prey in trial 1 in these two treatments (although these differences were not statistically significant Fig. 2a, d, trial 1; Tukey HSD: *p* = 0.23).

**Table 1:** Linear mixed effect model for log_10_-transformed time spent wrapping by *P. globosus* (Pholcidae) spiderlings using trial number, first-instar treatment, and their interaction as explanatory terms.

Fixed effects	D.F. num, den	*F*-value	*p*-value
Trial number	2, 133.4	0.86	0.42
First instar treatment	3, 25.53	7.03	0.0013*
Trial number x First instar treatment	6, 133.9	5.09	< 0.001*

Random effects			Wald *p*-value
Brood			0.24
Spider (Brood)			0.87

We only used data from trials along the first instar (trials 1–3) in this model. We adjusted our criterion for significance to α = 0.05/2 = 0.025 following the Bonferroni method since we used data from trial 3 in two statistical tests. We included brood and individual spiderling identity nested within brood as random effects in the model. Significant terms are designated by *.

Across the molt, there was an indication of overall improvement in prey capture efficiency (significant main term for trial number in Table 2; Fig. 2). However, whether improvement occurred also depended on the treatment (significant treatment and treatment x trial interaction terms in Table 2; Fig. 2). Specifically, spiderlings that received easy prey improved in efficiency (Fig. 2a, b), whereas spiderlings that received difficult prey did not (Fig. 2c, d). Interestingly, there was one treatment where spiderlings that received difficult prey after the molt improved in efficiency (Fig. 2d, trials 2 and 4). However, this is only true when comparing the efficiency of difficult prey to other difficult prey (marginally significant Tukey HSD: *p* = 0.057).

**Table 2:** Linear mixed effect model for log_10_-transformed time spent wrapping by *P. globosus* (Pholcidae) spiderlings using trial number, second-instar treatment, and their interaction as explanatory terms.

Fixed effects	D.F. num, den	*F*-value	*p*-value
Trial number	1, 45.91	5.19	0.027
Second instar treatment	3, 13.72	4.84	0.0017*
Trial number x Second instar treatment	3, 46.06	14.99	< 0.001*

Random effects			Wald *p* value
Brood			0.39
Spider (Brood)			0.17

We only used data from trials 3 and 4 in this model. We adjusted our criterion for significance to α = 0.05/2 = 0.025 following the Bonferroni method since we used data from trial 3 in two statistical tests. We included brood and individual spiderling identity nested within brood as random effects in the model. Significant terms are designated by *.

The post-hoc analyses suggest that the “steepness” of change in efficiency was the same regardless of whether the switch in prey type occurred before or across the molt. Changes in efficiency after a switch from difficult to easy prey were similar (trial *x* treatment interaction: F_1,_, _30.61_ = 2.86, *p* = 0.10, trials 3 and 4 in Fig. 2a, trials 2 and 3 in Fig. 2d) as were changes after a switch from easy to difficult prey (trial *x* treatment interaction: F_1,_, _34.54_ = 0.14, *p* = 0.71, trials 2 and 3 in Fig. 2c, trials 3 and 4 in Fig. 2d), regardless of timing.

## Discussion

We aimed to distinguish the effects of learning and maturation on prey capture efficiency in *P. globosus* spiderlings. For this, we varied the degree of familiarity and difficulty of prey spiderlings received and examined changes in efficiency in repeated trials within the spiderlings’ first instar and across the molt to the second instar. Overall, we found that the switch in prey type, rather than learning or maturation, primarily impacts the predatory behaviors of spiderlings.

Along the first instar, we found no overall improvement in efficiency based on prior experience. Instead, improvement depended on the type of prey spiderlings received: spiderlings were “more efficient” with easy prey rather than difficult prey. This suggests that the spiderlings did not use learning to improve their predatory behaviors along the first instar. We are confident that we gave spiderlings enough time to learn. There is evidence of learning to adjust web structure^21^, orient to prey faster^45^, and to avoid dangerous prey^46^ in as little as three trials in other spiders. We recorded spiderling behavior starting from their first prey-capture experience through their third. This should be ample time for learning to occur and has the potential for the largest opportunity most learning. The first few prey captures are crucial to ensure a spiderlings’ survival during these early vulnerable stages. Consequently, we argue that there would be strong selection on improving capture efficiency during that time. We note that in one prey-sequence treatment, spiderlings seemed to improve in efficiency after experience with difficult prey (Fig. 2d). We speculate one reason for this differential improvement could be the prey these spiderlings received may have struggled more than others, as individual prey can vary in how much they struggle during capture^17^. However, we did not quantify prey struggle. This speculation is based on the variation in time spiderlings spent wrapping difficult prey in trial 1 (although this variation not significant). Especially difficult prey may have offered the spiderlings a better opportunity for learning, as has been found in copepods^26^, fish^27^, and frogs^27^. Therefore, although prey difficulty primarily drove differences in prey capture efficiency along the first instar in our experiment, there may be some evidence for learning with especially difficult prey.

We also found no overall improvement in prey capture efficiency across the molt. Instead, we found that the type of prey spiderlings received primarily impacted efficiency. As there are many changes associated with maturation that can improve a spider’s ability to catch prey^7,47^, our results may suggest that the effect of maturation occurs over longer periods of time, rather than across just one molt. Similar results were found in *Misumena vatia* crab spiders, where spiderlings tested across a single molt, did not improve in the time required to capture prey^45^. Perhaps testing across additional molts might reveal an effect of maturation, but such tests would also have to account for additional confounding factors (e.g., variation in the duration of different instars^48^, disproportionate growth between individuals, effect of cumulate experiences). We note that there was one prey-sequence treatment where spiderlings seemed to improve with difficult prey across the molt, but only when comparing the efficiency with other difficult prey (Fig. 2d, trials 2 and 4). These spiderlings received difficult prey in three out of four trials and, as mentioned previously, we speculate that the prey these spiderlings received in trial 1 may have struggled more than others. Experience with such especially difficult prey may enhance the effects of maturation. Prey that struggle more may offer more opportunities for movements that enhance muscle growth, as with exercise. Although little is known about the effects of exercise in spiders, exercise has been shown to increase muscle development in mammals and insects^49^. In spiders, increased muscle mass aids prey capture^50,51^. Muscle development, therefore, could impact prey capture efficiency by aiding in handling and/or wrapping prey more effectively.

Here we found no evidence of learning or maturation regarding prey capture efficiency in *P. globosus* spiderlings. Nevertheless, we suggest our proposed heuristic and empirical criteria are effective at differentiating the effects of these processes. For example: if there was an overall improvement in efficiency along the first instar, this would have indicated an effect of learning. If there was an overall improvement in efficiency across the molt, this would have indicated an effect of maturation. However, this was not the case for our experiment. Although we did not find evidence for learning or maturation in spiderlings of this species, we believe our heuristic will be broadly useful at differentiating these processes in the future. It would be interesting to apply our heuristic with other animal taxa, specifically those with discrete maturational events, to investigate the roles of learning and maturation on other behaviors.

### Supplementary Information

Supplementary Information.

## Data Availability

The data analyzed in this study are available in the supplementary materials.
